# Transactions of the Southern Surgical and Gynecological Association, Vol. VI

**Published:** 1894-07

**Authors:** 


### Book Reviews.

TRANSACTIONS OF THE SOUTHERN SURGICAL AND GYNECOLOGICAL ASSOCIATION. Vol. VI. Sixth Session, held at New Orleans, La., November 14, 15, 16, 1893. Published by the Association. 1894.

It is only necessary to mention the names of a few members who contributed papers at the last meeting of this Association to prove the great worth of these transactions, viz.: Dr. Bedford Brown, Howard A. Kelly, Joseph Price, Geo. J. Englemann, Willis F. Westmoreland, Hunter McGuire, J. McFadden Gaston, and many others of no less repute.

The Association is composed of men who stand the highest in surgery and gynecology, Dr. E. H. Sholl, speaking in glowing terms of the growth and standing of the Association, says: "I stand with amazement as I see before me so many brilliant men, who have taken the deepest possible interest in this Association. One of its beautiful and striking features is, that it ignores any limitation of latitude. . . . It is recognized at all times and on all occasions, and characterized by the unity and harmony of our glorious profession, full orbed in its type and vigorous manhood. Already in its sixth year of existence, it is not only a recognized factor in the South, but it meets with the approval of our distinguished brethren of the North and West, who are participating in its proceedings, who are helping to bring it to the front, making it one of the greatest Associations, of its kind, in the United States; I say this, unhesitatingly, because its worth has been recognized both at home and abroad."

These volumes, being composed of the best writings and discussions of these scientific men who are doing so much to adorn medical science in America, are simply invaluable to all who are the least interested in the progress of surgery and gynecology. Space forbids further mention of the excellency of this book.

